# Influenza A(H1N1)pdm09-Associated Pneumonia Deaths in Thailand

**DOI:** 10.1371/journal.pone.0054946

**Published:** 2013-02-04

**Authors:** Charatdao Bunthi, Somsak Thamthitiwat, Henry C. Baggett, Pasakorn Akarasewi, Ruchira Ruangchira-urai, Susan A. Maloney, Kumnuan Ungchusak

**Affiliations:** 1 International Emerging Infections Program, Global Disease Detection Regional Center, Thailand Ministry of Public Health- US Centers for Disease Control and Prevention Collaboration, Nonthaburi, Thailand; 2 Bureau of Epidemiology, Thailand Ministry of Public Health, Nonthaburi, Thailand; 3 Department of Pathology, Siriraj Hospital, Mahidol University, Bangkok, Thailand; The University of Hong Kong, China

## Abstract

**Background:**

The first human infections with influenza A(H1N1)pdm09 virus were confirmed in April 2009. We describe the clinical and epidemiological characteristics of influenza A(H1N1)pdm09-associated pneumonia deaths in Thailand from May 2009-January 2010.

**Methods:**

We identified influenza A(H1N1)pdm09-associated pneumonia deaths from a national influenza surveillance system and performed detailed reviews of a subset.

**Results:**

Of 198 deaths reported, 49% were male and the median age was 37 years; 146 (73%) were 20–60 years. Among 90 deaths with records available for review, 46% had no identified risk factors for severe influenza. Eighty-eight patients (98%) received antiviral treatment, but only 16 (18%) initiated therapy within 48 hours of symptom onset.

**Conclusions:**

Most influenza A(H1N1)pdm09 pneumonia fatalities in Thailand occurred in adults aged 20–60 years. Nearly half lacked high-risk conditions. Antiviral treatment recommendations may be especially important early in a pandemic before vaccine is available. Treatment should be considered as soon as influenza is suspected.

## Introduction

The 2009 influenza pandemic virus, influenza A(H1N1)pdm09 was first confirmed in the United States by the Centers for Disease Control and Prevention (CDC) in April 2009 and rapidly spread worldwide [1], [2], [3], [4]. Clinical manifestations of influenza A(H1N1)pdm09 infection ranged from mild symptoms to severe illness and death. Most patients with severe or fatal disease were reported to have underlying medical conditions, including chronic lung disease, diabetes, cardiovascular disease, neurological disease, and pregnancy [5], [6], [7], [8].

The first two cases of laboratory-confirmed influenza A(H1N1)pdm09 infection in Thailand were reported on May 10, 2009, in exchange students who returned from Mexico. Although the epidemiology of influenza A(H1N1)pdm09 deaths has been well-described in the United States, Mexico, and Europe [1], [9], [10], less is known about fatal cases in Thailand or other countries in Asia [11], [12], [13].

We present epidemiological and clinical data on influenza A(H1N1)pdm09-associated deaths among persons hospitalized with pneumonia in Thailand, collected through retrospective review of medical records.

### Ethical Considerations

The medical records reviews were considered by the MOPH to be part of the public health response to the 2009 influenza pandemic in Thailand and therefore did not require review by the human subjects Ethical Review Committee.

## Methods

In 2004, Thailand’s Ministry of Public Health (MOPH) established the National Avian Influenza Surveillance (NAIS) system in response to human cases of avian influenza A(H5N1). Under NAIS, hospitals were required to report all cases of severe and fatal human influenza infection to the Bureau of Epidemiology (BOE) [14]. In May 2009, at the start of the influenza A(H1N1)pdm09 outbreak in Thailand, the MOPH encouraged reporting of all suspected influenza A(H1N1)pdm09 cases through the NAIS system along with submission of respiratory specimens (nasopharyngeal swabs, throat swabs, or endotracheal tube aspirates) to be tested at Thailand’s National Institute of Health (NIH) for influenza viruses by real-time reverse transcription polymerase chain reaction (rRT-PCR). Reports to NAIS were submitted by hospital epidemiologists (or clinicians) electronically through a web-based system or using paper forms and included information on patient demographics, underlying medical conditions, clinical characteristics, and outcome.

In addition, MOPH established a parallel surveillance system in early 2009 to support the investigation of severe and fatal pneumonia cases. Due to the natural overlap with NAIS, cases from this severe and fatal pneumonia surveillance system were reported through NAIS, but additional data and specimens were requested. Under the severe and fatal pneumonia surveillance system, clinicians were encouraged to submit respiratory specimens and blood (serum or whole blood) for testing from patients with community-acquired pneumonia that required intubation and did not respond to treatment within 48 hours or resulted in death. Clinicians were also encouraged to collect post-mortem tissue specimens from fatal cases through consented autopsy or necropsy. Respiratory, blood, and fresh frozen tissue specimens in sterile containers were kept and shipped to the NIH for virology and bacteriology testing within 48 hours. Formalin-fixed tissue specimens were sent to the Department of Pathology at Siriraj Hospital in Bangkok, where they were embedded in paraffin, cut into 3 µm-thick sections, deparaffinized in xylene, and rehydrated in graded alcohol. Each section was stained with hematoxylin and eosin. During the influenza A(H1N1)pdm09 pandemic, under the auspices of this surveillance system, MOPH also requested that hospitals make medical records of fatal influenza A(H1N1)pdm09-associated pneumonia cases available for review by public health officials.

For this investigation, efforts were made to acquire medical records from hospitals for all cases of influenza A(H1N1)pdm09-associated pneumonia deaths that occurred during the first wave and the beginning of second wave of the pandemic in Thailand (May 2009 through January 2010), but records for only 90 cases were available. Data on demographics, medical history, clinical course, laboratory testing, and treatment were abstracted by trained clinicians, nurses, and epidemiologists using a standardized form. All chest radiographs (CXR) for which hospital radiology reports were not available were reviewed by a radiologist from the MOPH Chest Disease Institute.

### Data Analysis

We first present descriptive statistics for all fatal cases of influenza A(H1N1)pdm09-associated pneumonia reported to Thailand’s NAIS system from May 2009 through January 2010, defined as any death in a patient hospitalized with clinician-diagnosed pneumonia and at least one respiratory specimen positive for influenza A(H1N1)pdm09 by rRT-PCR. More detailed descriptions of clinical characteristics, treatment, and outcomes are presented for the subset of cases for which medical record reviews were performed.

Descriptive data are presented as frequencies for discrete variables and as means or medians for continuous variables. SPSS version 17.0 (SPSS Inc, Chicago, Illinois) was used for all analyses. Underlying medical conditions considered high-risk for severe influenza infection were based on the World Health Organization’s document, “Clinical Management of Human Infection with Pandemic (H1N1) 2009 Virus”: chronic pulmonary disease (including asthma and chronic obstructive pulmonary disease (COPD)), cardiovascular disease (except hypertension alone), metabolic disorders (including diabetes mellitus), chronic renal disease, certain neurological conditions, immunosuppression (e.g., HIV, cancer), pregnancy and obesity. Body mass index (BMI) was calculated as weight in kilograms divided by a square of height in meters (kg/m2) when measurements were available in the medical records; obese and morbidly obese were defined as a BMI of 30–39 kg/m^2^ and a BMI of ≥40 kg/m^2^, respectively [15].

## Results

**Figure 1:**
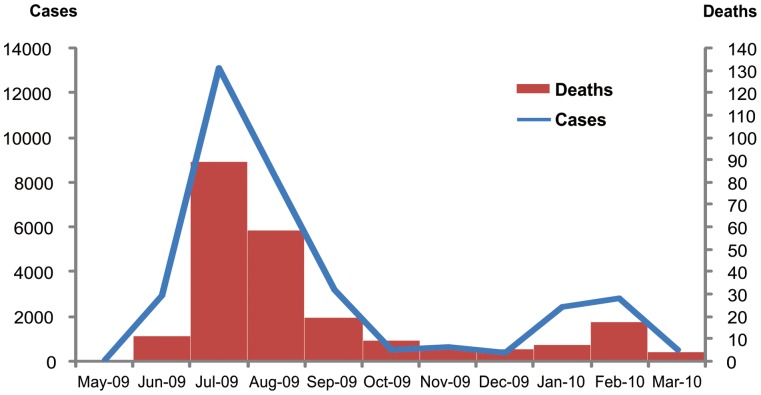
Laboratory-confirmed influenza A (H1N1)pdm09 cases and deaths. Detailed legend: Laboratory-confirmed influenza A (H1N1)pdm09 cases and deaths reported to Bureau of Epidemiology, Ministry of Public Health Thailand from May 2009-March 2010. Bars represent number of deaths. Line represents number of cases.

**Figure 2:**
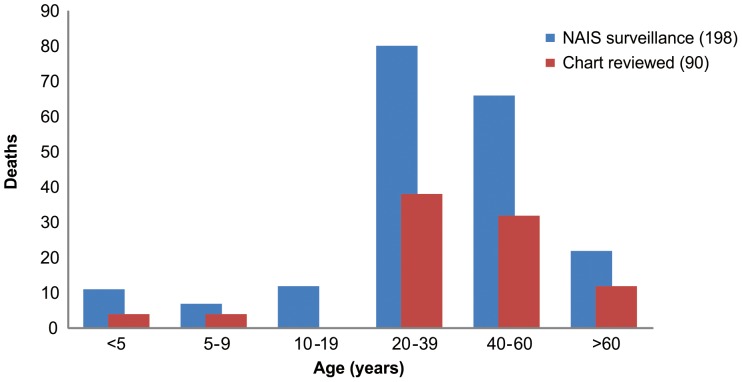
Age distribution of influenza A (H1N1)pdm09 deaths. Detailed legend: Age distribution of influenza A (H1N1)pdm09 deaths reported to the National Avian Influenza Surveillance (NAIS) system, Bureau of Epidemiology, Ministry of Public Health, Thailand and those for whom medical charts were available for review - May 2009-January 2010. Bars represent number of deaths for each age group (Figures are in a separate file).

From May 2009 through January 2010, 27,254 cases and 198 fatal cases of laboratory-confirmed influenza A(H1N1)pdm09-associated pneumonia were reported to the MOPH-NAIS system (0.7% case fatality proportion) (Figure 1). The median age for all reported cases was 37 years (range, 0 to 91) and 49% were male (Table 1, Figure 2). Data on underlying medical conditions were available for 130 (66%) patients, and 68% were reported to have at least one known high-risk condition for severe influenza infection. Although obesity was noted as a co-morbid condition in 22 (17%) cases, weight and height data to confirm the diagnosis were available for only 14 cases, 9 (64%) of whom had BMI ≥30 kg/m^2^.

**Table 1:** Demographic characteristics and underlying medical conditions of influenza A (H1N1)pdm09 virus-associated pneumonia fatalities in Thailand, May 2009-January 2010.

Characteristic	No. (%)Total cases = 198	No. (%)Medical records reviewed = 90
Male	97 (49)	50 (56)
Age group (years)		
Age <5	11 (6)	4 (4)
5–9	7 (4)	4 (4)
10–19	12 (6)	0 (0)
20–39	80 (40)	38 (42)
40–60	66 (33)	32 (36)
>60	22 (11)	12 (13)
**High risk condition for severe influenza** * **N = 130 N = 90**		
Diabetes Mellitus	25 (19)	16 (18)
Cardiovascular diseases	13 (10)	10 (11)
Kidney disease†	11 (8)	8 (9)
Pregnancy	11 (8)	5 (6)
Obesity‡	9/14 (64)	6/13 (46)
Asthma	8 (6)	5 (6)
Chronic lung disease§	10 (8)	5 (6)
Cancer	6 (5)	5 (6)
HIV	2 (2)	2 (2)
Neuromuscular diseases	9 (7)	1 (1)
Thalassemia	2 (2)	1 (1)
At least one of the above conditions	89 (68)	49 (54)
Not report for the above conditions	41 (32)	41 (46)

* According to WHO on Clinical Management of Human Infection with influenza A(H1N1)pdm09 [15].

† Kidney disease includes chronic renal failure, polycystic kidney disease.

‡ Obesity defined as body mass index (BMI) ≥30 kg/m^2^. BMI calculated as weight in kilograms divided by height in meters squared among non-pregnant patients; 14 patients overall and 13 patients with medical records reviewed had height and weight available BMI calculation.

§ Chronic lung disease includes obstructive pulmonary disease, chronic bronchitis, and pulmonary tuberculosis.

Medical records were available for review for 90 (45%) of 198 reported influenza A(H1N1)pdm09-associated pneumonia deaths. The median age of these 90 patients was 38.5 years (range, 0–91) and 56% were male (Table 1, Figure 2). Of the 90 fatal cases with records reviewed, 49 (54%) patients had evidence of at least one known high-risk condition for severe influenza, most commonly diabetes mellitus (18%); 42 (47%) patients had no identified risk factors. Of the 13 non-pregnant patients with height and weight available for BMI calculation, three (23%) were obese (BMI of 30–39 kg/m^2^) and 4 (31%) were morbidly obese (BMI ≥40 kg/m^2^).

Among the 90 fatal cases with medical record reviews completed, none had received the monovalent influenza pandemic strain vaccine but one (1%) had received seasonal influenza vaccine. Five cases (6%) occurred among pregnant women and all five were treated with oseltamivir. Three were in the second trimester of pregnancy, and all three experienced fetal demise. Two patients were in the third trimester and both delivered their babies by caesarian section. One of the two neonates, delivered after 31 weeks gestation weighing 1,560 grams, was diagnosed with laboratory-confirmed influenza A(H1N1)pdm09 infection on the first day of life. This baby was treated with oseltamivir, recovered and was discharged at 28 days of age [13]. The other baby, who was delivered at 28 weeks gestation weighing 1,230 grams, developed respiratory distress syndrome (rRT-PCR of respiratory specimen was negative for influenza A(H1N1)pdm09), and died after 12 days.

Sixty-one patients (68%) had sought medical care at least once as an initial visit at local health care facilities (local health center, private clinic, private or public hospital) before being hospitalized. This includes 57 patients who were hospitalized at another facility before the hospitalization during which death occurred, hereafter referred to as the final hospitalization.

At the final hospitalization, 17% had influenza-like illness (documented fever ≥38°C with cough or sore-throat). Forty-six percent of patients did not have documented fever at admission. Based on patient histories, 73% had report of fever with cough or sore throat. Of 88 patients with documentation of a CXR, 62 (69%) had either CXR reports or films available for radiologist review. All 62 patients had abnormal CXR; 53 (85%) patients showed diffuse pulmonary infiltration and 9 (15%) patients had localized pulmonary infiltration. Seventy-seven (86%) patients were managed in an intensive care unit, 89 (99%) received mechanical ventilation, and 54 (60%) were diagnosed with acute respiratory distress syndrome. Antibiotic therapy was prescribed to all patients except one during the first five days of the final hospitalization, and 37 (42%) patients received steroids. Eight (9%) patients had chronic renal failure requiring dialysis (Table 2).

**Table 2:** Hospital course of 90 influenza A (H1N1)pdm09 virus-associated pneumonia deaths in Thailand for whom medical record reviews were conducted, May 2009-January 2010.

Hospital Course	No. (%)
Admission to intensive care unit	77 (86)
Mechanical ventilation	89 (99)
Positive blood culture*	3 (3)
Shock requiring vasopressor therapy	80 (89)
Acute Respiratory Distress Syndrome	54 (60)
Renal failure with dialysis	8 (9)
Antiviral treatment	88 (98)
Oseltamivir	88 (98)
Zanamivir	9 (10)
Antibiotic treatment within first 5 days of admission	89 (99)
Received steroid treatment during hospitalization	37 (42)
Leukopenia (WBC <5,000)	28 (31)
Leukocytosis (WBC >15,000)	21 (23)
Hemoglobin <10	12 (13)
Platelet count <100,000‡	11 (12)
Serum creatinine >1.5§	25 (29)
AST >2xUNL (70)¶	29 (57)
ALT >2xUNL (80)¶	17 (33)

* Blood culture positive for *Acinetobacter baumannii* (1), *Salmonella* group D and *pseudomonas* spp. (1) and *Staphylococcus aureus* (1).

** Records available for 89 of 90 patients.

*** Records available for 85 of 90 patients.

**** Records available for 51 of 90 patients, AST (aspatate aminotransferase), ALT (alanine transaminase); UNL (upper normal limit).

Antiviral treatment was administered to 88 (98%) patients, all of whom received oseltamivir. Zanamivir was used in combination with oseltamivir for nine patients. Oseltamivir was initiated during the final hospitalization for 87 patients, while only one patient had received treatment at a previous medical facility (3 days before the final hospitalization). The median time from symptom onset to the first dose of oseltamivir was 4.5 days (range, 0 to 20 days). The median time from symptom onset to death was 9 days (range, 1 to 46 days). The median time from admission to death and from first dose of oseltamivir to death was 4.5 days and 4 days (range, 0 to 32 days for both), respectively (Table 3). The timing between each step in the clinical course between symptom onset, hospitalization, oseltamivir administration, and death was similar for patients aged <18 years and those ≥18 years, except the time from symptom onset to the first dose of oseltamivir was significantly shorter among patients aged 18 years and older (p = 0.01).

**Table 3:** Timing of antiviral therapy relative to clinical course of 90 influenza A (H1N1)pdm09 virus-associated pneumonia fatalities in Thailand for whom medical record reviews were conducted, May 2009-January 2010.

Clinical course	Median time in Days (Range)	p-Value*
Symptom onset to hospital admission	4.0 (0–14)	
Age <18 years (n = 8)	5.0 (2–14)	0.31
Age ≥18 years (n = 82)	4.0 (0–14)	
Symptom onset to 1st Oseltamivir dose	4.5 (0–20)	
Age <18 years (n = 7)	8.0 (3–14)	0.01
Age ≥18 years (n = 81)	4.0 (0–20)	
Hospital admission to 1st Oseltamivir dose	0.0 (−3–10)	
Age <18 years (n = 7)	1.0 (0–9)	0.09
Age ≥18 years (n = 81)	0.0 (−3–10)	
Symptom onset to death	9.0 (1–46)	
Age <18 years (n = 8)	13.5 (4–46)	0.43
Age ≥18 years (n = 82)	9.0 (1–25)	
Hospital admission to death	4.5 (0–32)	
Age <18 years (n = 8)	6.5 (1–32)	0.31
Age ≥18 years (n = 82)	4.5 (0–18)	
First Oseltamivir dose to death	4.0 (0–32)	
Age <18 years (n = 7)	1.0 (0–32)	0.94
Age ≥18 years (n = 81)	4.0 (0–18)	

* Mann-Whitney U test comparing age <18 to ≥18 years.

Nine patients (10%) had blood culture results available; all blood cultures were done on the first day of the final hospitalization. Three (33%) were positive for possible pathogens: *Acinetobacter baumannii* (1), *Salmonella* group D and *Pseudomonas* spp. (1), and *Staphylococcus aureus* (1). All three patients with positive cultures had been previously admitted to another hospital and were transferred within 24 hours of the original admission.

Pulmonary tissue was submitted for 12 patients, but only five specimens were adequate for histopathological examination. Diffuse alveolar damage was found in two patients; the other three were found to have focal lymphocytic interstitial infiltrates, pulmonary congestion and pulmonary edema, respectively. Six of the 12 patients with pulmonary tissue collected had fresh frozen specimens submitted for bacterial PCR testing at the Thailand NIH using in-house assays, all of which were negative for *Chlamydophila pneumoniae*, *Mycoplasma pneumoniae*, *Legionella* species, *Streptococcus pneumoniae*, *Haemophilus influenzae*, *Moraxella catarrhalis*, *Burkholderia pseudomallei*, *Escherichia coli*, *Klebsiella pneumoniae*, *Pseudomonas aeruginosa*, *Stenotrophomonas maltophilia* and *Acinetobacter* species.

## Discussion

We found that the majority of influenza A(H1N1)pdm09-associated pneumonia deaths in Thailand occurred in adults aged 20 to 60 years, which is similar to previous studies [16], [17], but differed from the older age predominance of seasonal influenza deaths [18], [19]. Only 53% of fatal cases with medical records reviewed had evidence of high-risk conditions for severe influenza infection, although the proportion was higher (67%) among all 198 fatal cases reported to NAIS. Regardless, the proportion of fatal cases with no reported high-risk condition was substantial and was on the lower end of what has been reported in other countries. The prevalence of high-risk conditions among fatal influenza A(H1N1)pdm09 cases in the U.S. ranged from 68% [20] to 73% [5] and in Brazil was 55% (excluding obesity) [21]. Similar to previous studies [5], [22], [23], [24], [25], [26], [27], [28], [29], [30], diabetes mellitus, cardiovascular disease and chronic lung disease were common underlying medical conditions among influenza A(H1N1)pdm09 virus-associated deaths in Thailand.

Oseltamivir was administered to nearly all patients in our report, which was likely facilitated by domestic production in Thailand and by efforts to preposition antiviral medication in hospitals around the country. The antiviral treatment stockpile in Thailand was sufficient to treat 0.5% of the population at the beginning of the pandemic, with one million treatment courses added during the first wave of the pandemic [31]. However, treatment was initiated within 48 hours of symptom onset in only 16 (18%) patients. Further, antiviral treatment was almost never prescribed to patients on their initial visit to a health care facility. Studies have shown that early oseltamivir treatment (<48 hours after symptom onset) is associated with decreased risk of ICU admission and death [5], [14], [32]. The median duration from illness onset to initiation of antiviral therapy was 4.5 days, well beyond the 48 hour time period recommended by WHO and the U.S. CDC [15], [33]. Our finding of delayed antiviral initiation among fatal influenza A(H1N1)pdm09 cases was similar to findings reported from the U.S., Mexico and China [5], [8], [25] and may have been related to insufficient information disseminated to clinicians early in the outbreak, resulting in lack of clinical recognition of influenza, lack of familiarity with recommendations for empiric antiviral therapy [15], or concern over the potential development of drug resistance. Anecdotal reports also indicated that clinicians may have been reluctant to prescribe antiviral drugs without laboratory confirmation of influenza virus infection; laboratory testing for influenza viruses is not frequently available in the outpatient setting in Thailand.

Our report is subject to several limitations. Although 198 hospitalized influenza A(H1N1)pdm09-associated pneumonia fatalities were identified, medical records were only available for review for 90 (45%). Additionally, some influenza A(H1N1)pdm09 deaths likely occurred outside of the hospital [34]. Therefore, our findings may not be representative of all hospitalized influenza A(H1N1)pdm09 pneumonia deaths or of fatal influenza A(H1N1)pdm09 cases in Thailand overall. However, the age and sex distribution, as well as the prevalence of most high-risk conditions, for the 90 cases in our series was similar to that of all 198 fatal influenza A(H1N1)pdm09 pneumonia cases reported to the NAIS system. Data were extracted solely from chart review, limiting results to recorded data and prohibiting verification by interview with relatives or clinicians. As a result, we may have underestimated the number of patients with underlying medical conditions, despite efforts made to review both inpatient and outpatient records from all health facilities where the patient received treatment. Height and weight were available for very few patients, preventing a robust assessment of obesity prevalence, a putative risk factor for severe influenza infection [6], [15], [21], [25]. Finally, we lacked a comparison group of hospitalized non-fatal influenza A(H1N1)pdm09 pneumonia cases so were unable to determine the proportion of hospitalized patients who died or assess risk factors for death. Although we were not able to assess risk factors, other studies conducted during the pandemic confirmed that many of the WHO-defined risk factors [15] did increase the risk of severe disease and death among persons infected with influenza A(H1N1)pdm09 [6], [20], [24], [35].

Despite these limitations, our findings that the majority (78%) of influenza A(H1N1)pdm09 virus-associated fatalities were in adults aged 20–60 years and that one-third to one-half had no known high-risk medical conditions may have implications for public health practice in Thailand. Consistent with early guidance from the World Health Organization’s Strategic Advisory Group of Experts in July 2009, pandemic vaccine recommendations in Thailand initially included healthcare workers, pregnant women and persons with high-risk medical conditions and were soon expanded to include children aged 6 months to 2 years [36]. Although Thailand did not recommend vaccination for healthy adults [37], the high proportion of deaths observed in this group suggests that a wider vaccination strategy may have been beneficial if resources had allowed. However, the monovalent pandemic vaccine was not widely available in Thailand until January 2010, after our review was completed. During the pandemic, Thailand also had targeted recommendations in place for seasonal influenza vaccine, which has been suggested in at least one report to have provided some protection against influenza A(H1N1)pdm09 [38], but uptake was generally low [39]. In countries without early access to pandemic vaccines, antiviral treatment recommendations may ultimately be more important. Antiviral treatment should be considered as soon as influenza is suspected, especially in patients with high-risk condition [33]. To improve clinical outcomes, interventions to facilitate earlier antiviral administration should be pursued. Further evaluation of the entire spectrum of influenza A(H1N1)pdm09 virus-associated illness would allow better estimation of the burden of the 2009 pandemic in Thailand and may help identify risk factors for severe illness and guide prevention and control efforts during seasonal epidemics and for future pandemics.
